# Orchestrating immunopathology: the spectrum of programmed cell death pathways co-opted by influenza a virus in pulmonary immunity

**DOI:** 10.3389/fimmu.2026.1705500

**Published:** 2026-01-29

**Authors:** Yuanyuan Luo, Li Xiang, Qingman He, Yongxiang Gao, Chuan Zheng, Huan Yao

**Affiliations:** 1 School of Clinical Medicine, Hospital of Chengdu University of Traditional Chinese Medicine, Chengdu, China; 2 School of Clinical Medicine, Chengdu University of Traditional Chinese Medicine, Chengdu, China; 3 Sichuan Provincial Engineering Technology Research Center of Natural Small Molecule Drug, Tianfu Traditional Chinese Medicine (TCM) Innovation Harbour, Chengdu University of Traditional Chinese Medicine, Chengdu, China; 4 Traditional Chinese Medicine (TCM) Regulating Metabolic Diseases Key Laboratory of Sichuan Province, Hospital of Chengdu University of Traditional Chinese Medicine, Chengdu, China; 5 Sichuan Provincial Engineering Research Center of Innovative Re-Development of Famous Classical Formulas, Tianfu Traditional Chinese Medicine (TCM) Innovation Harbour, Chengdu University of Traditional Chinese Medicine, Chengdu, China

**Keywords:** immune homeostasis, influenza A virus, programmed cell death, respiratory epithelial barrier, virus-host interactions

## Abstract

Influenza A virus (IAV) infection activates multiple programmed cell death (PCD) pathways, which, while restricting viral replication and dissemination, concurrently disrupt the respiratory epithelial barrier and compromise immune homeostasis. Excessive activation of apoptosis, necroptosis, pyroptosis, and related processes results in tight junction(TJ) disruption, impaired mucociliary clearance and gas exchange, and amplification of inflammatory cascades, ultimately driving cytokine storm and severe tissue injury. This dual role of PCD underscores its importance in antiviral defense while exposing its potential to exacerbate immunopathology. Accordingly, this review focuses on IAV-induced PCD mechanisms, delineating their contribution to epithelial barrier breakdown and immune dysregulation, with the aim of informing strategies for precise modulation of immunopathological damage and improving therapeutic outcomes in severe influenza.

## Introduction

Influenza viruses belong to the family *Paramyxoviridae* and are enveloped viruses with segmented negative-sense RNA (nsRNA) genomes. The genome comprises eight single-stranded RNA segments encapsidated by nucleoproteins (NP) and encodes at least 12 structural and non-structural proteins, including the polymerase subunits PA, PB1, and PB2; hemagglutinin (HA); neuraminidase (NA); matrix proteins (M1, M2); and non-structural proteins (NS1, NS2) (1). Among them, influenza A virus (IAV) is the most common type of clinical infection and exhibits a broad host range, including humans, swine, bats, and various wild birds (2). Due to its pronounced disease burden and significant public health impact, IAV is the principal focus of this review.

## Overview of the IAV life cycle, tissue tropism, and immune evasion mechanisms

IAV infection begins with the binding of viral HA to sialic acid (SA) receptors on the surface of respiratory epithelial cells (1). Human IAV preferentially recognizes the α2,6-linked galactose (SAα2,6Gal) receptor, which is predominantly distributed on ciliated epithelial cells of the upper respiratory tract, whereas alveolar epithelial cells of the lower respiratory tract mainly express α2,3-linked galactose (SAα2,3Gal) (3). This spatial difference in receptor distribution explains the predominance of upper respiratory tract infection by IAV. Further investigations have revealed that IAV strains, such as PR/8, display marked tropism toward distal lung epithelial stem/progenitor cells, with infection rates exceeding those observed in alveolar and small airway epithelial cells (4).

Following entry via clathrin-mediated endocytosis, the virus releases viral ribonucleoprotein complexes (vRNPs) into the cytoplasm, which are subsequently transported into the nucleus to initiate RNA transcription and replication (5). The RNA-dependent RNA polymerase of influenza A virus (IAV) exhibits intrinsically low replication fidelity, resulting in a high mutation rate that drives both antigenic drift and antigenic shift, thereby undermining host adaptive immune responses (6). Antigenic drift is characterized by the progressive accumulation of amino acid substitutions in the hemagglutinin (HA) and neuraminidase (NA) proteins—particularly within the functional domains of the HA globular head—leading to alterations in key antigenic epitopes. These changes impair the recognition of variant viruses by pre-existing strain-specific antibodies and memory T cells elicited by prior infection or vaccination (7). In contrast, antigenic shift arises from genomic reassortment events that generate antigenically novel viral subtypes, most commonly occurring during co-infection of a host with distinct IAV strains, thereby resulting in widespread population susceptibility due to the absence of pre-existing immune memory (8). Both mechanisms facilitate immune evasion by remodeling the antigenic architecture of HA and NA, ultimately promoting sustained viral circulation.

Beyond reliance on efficient genome replication, IAV employs multilayered regulatory strategies to attenuate host immune clearance during its replication cycle. In addition to escaping adaptive immunity through antigenic variation, IAV actively suppresses innate immune responses during viral replication and assembly, thereby further enhancing viral fitness and persistence within the host. The non-structural protein 1 (NS1) of IAV suppresses interferon signaling by blocking the activation of retinoic acid-inducible gene I (RIG-I) and interferon regulatory factor 3 (IRF3) (9), while PB2 interferes with the function of mitochondrial antiviral signaling protein (MAVS) to inhibit type I interferon expression (10). Moreover, IAV can acquire additional potential glycosylation sites on the hemagglutinin (HA) protein, thereby physically masking ligands recognized by natural killer (NK) cells. Concurrently, IAV induces conformational alterations in major histocompatibility complex class I (MHC I) molecules on the surface of infected cells, enhancing their interaction with inhibitory receptors such as killer cell Ig-like receptor 2 domain long tail 1(KIR2DL1) and leukocyte Ig-like receptor-1(LIR-1) (11, 12). Through this dual mechanism—attenuation of activating signals and reinforcement of inhibitory signaling—IAV effectively suppresses NK cell–mediated cytotoxicity, thereby further promoting viral survival and expansion within the host.

Within this immunoregulatory context, efficient viral release becomes a critical determinant of infection spread once viral replication and assembly are completed. These mechanisms collectively enable the virus to evade host innate immune responses. Upon completion of viral assembly, NA cleaves SA residues on the host cell surface to promote the release of progeny virions and their spread to neighboring cells, whereas proteolytic cleavage of HA by host proteases such as transmembrane serine protease 2 (TMPRSS2) is required for efficient viral release (13).

Collectively, these coordinated processes dynamically shape the balance among viral replication, host immune responses, and immune-mediated tissue pathology, thereby providing an integrated framework for understanding host–virus immunopathological interactions.

## Clinical relevance of IAV-induced cellular damage and immunopathology

**Figure 1:**
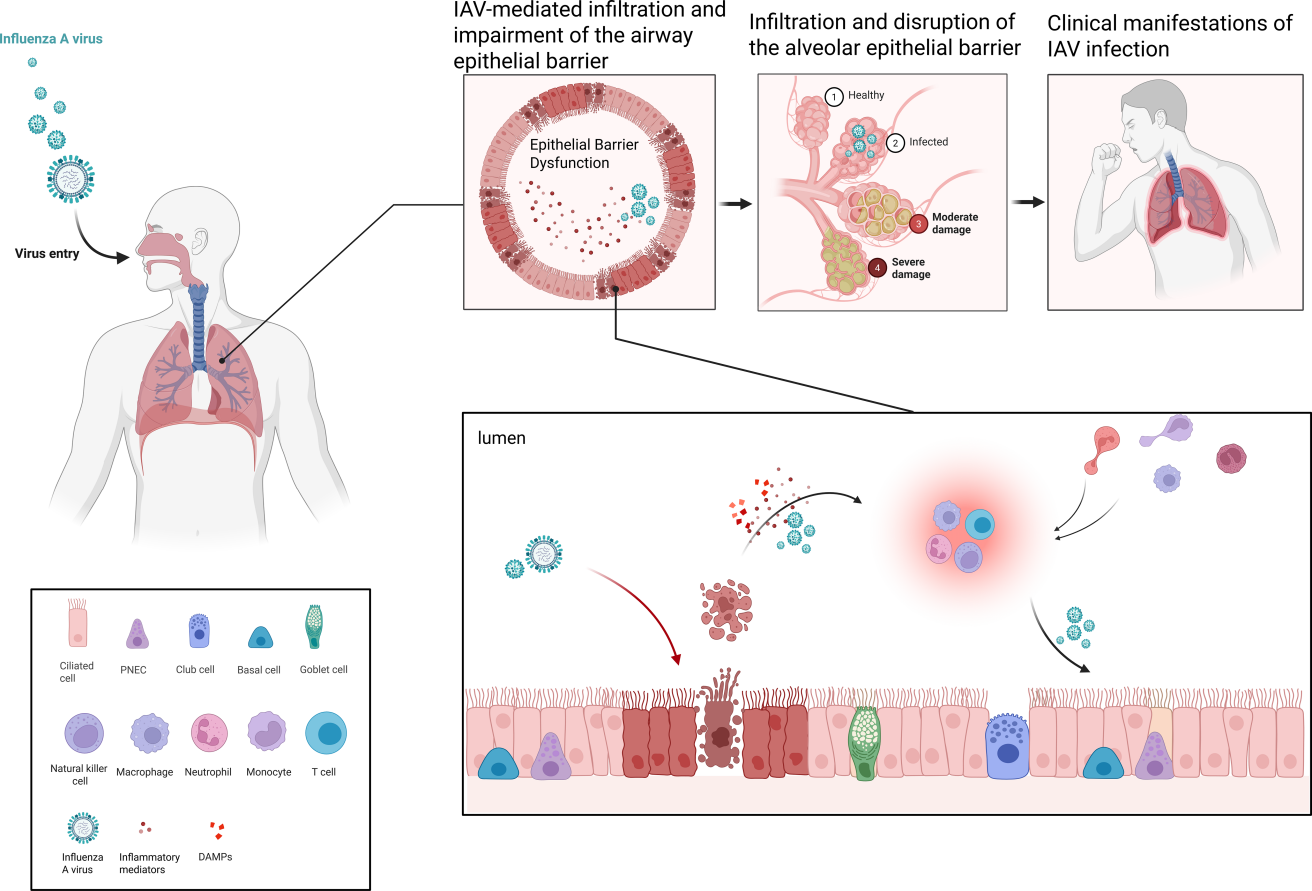
The respiratory epithelium constitutes the first line of defense against IAV invasion, and its structural and functional integrity is essential for restricting viral replication and spread. However, IAV infection often induces PCD, leading to epithelial injury. While PCD contributes to the elimination of infected cells and prevents further viral dissemination, it is simultaneously accompanied by the release of DAMPs, which amplify innate immune activation and drive excessive recruitment and activation of inflammatory cells. The persistence of such inflammatory responses disrupts the local tissue microenvironment, further compromising the integrity of both the alveolar epithelium and the capillary endothelium. This breakdown of the alveolar–capillary barrier results in pulmonary edema and impaired gas exchange, ultimately manifesting clinically as dyspnea, hypoxemia, and disease progression. Created in BioRender. Yuanyuan, Luo. (2026) https://BioRender.com/fqin2jp.

The tissue tropism and immune evasion strategies of influenza A virus (IAV) not only determine the initial site of infection and viral load but also establish the foundation for subsequent cellular injury and immunopathological responses. The detailed process is shown in **Figure 1**. Selective infection of distinct respiratory epithelial cell subsets results in differential patterns of local damage, thereby shaping the activation modes of the host immune system. By delaying interferon-mediated clearance, IAV allows early sustained replication within infected cells, thereby increasing the risk of injury to both infected and neighboring epithelial cells. This process triggers multiple programmed cell death (PCD) pathways, generating a dynamic balance between viral clearance and immunopathology.

Virus-induced cell death leads to the loss of both infected and adjacent cells while releasing large amounts of damage-associated molecular patterns (DAMPs, such as High Mobility Group Box 1(HMGB1), Adenosine Triphosphate (ATP), and mitochondrial DNA(mtDNA)) and pathogen-associated molecular patterns (PAMPs) (14), which continuously activate macrophages, dendritic cells, and neutrophils. Through Toll-like receptors (TLRs) and NOD-like receptors (NLRs), immune cells recognize these signals and activate NF-κB and inflammasome pathways, resulting in the production of large quantities of proinflammatory cytokines (IL-1β, IL-18, IL-6, TNF-α) and chemokines, thereby driving cytokine storm formation (15). IL-1β and IL-18 can be released through Gasdermin D (GSDMD) membrane pores or via non-classical routes such as exosomes and lysosomal exocytosis, further recruiting neutrophils and NK cells to amplify the inflammatory cascade (16).

In pulmonary tissue, extensive epithelial and immune cell death disrupts the alveolar–capillary barrier, leading to edema and impaired gas exchange (17). Excessive neutrophil activation results in the formation of neutrophil extracellular traps (NETs), which can capture viruses but also exacerbate endothelial injury and microthrombosis (18). NK cells, driven by IL-18, enhance IFN-γ secretion, further promoting immune cell infiltration and tissue damage (19). Moreover, high-risk populations, including obese and elderly individuals, are more prone to severe immunopathological responses due to impaired interferon signaling or immunosenescence (20). Collectively, IAV, through its viral life cycle, tissue tropism, and immune evasion strategies, together with the interplay of PCD and immune signaling, not only facilitates viral clearance but also significantly contributes to pulmonary tissue damage and disease severity.

## Defense and disruption mechanisms of the respiratory epithelial barrier in IAV infection

The respiratory epithelium(The relevant information regarding the cellular composition of this structure is provided in **Table 1**), as the primary defense line against pathogen invasion, establishes a dual physical barrier through the mucus–ciliary clearance system and tight junction proteins (TJs), thereby maintaining mucosal microenvironmental homeostasis. The mucus layer, mainly composed of mucin 5AC (Muc5AC) and mucin 5B (Muc5B) secreted by goblet cells, determines the rheological properties of mucus, which provide the structural basis for cilia-mediated clearance (21). Coordinated rhythmic beating of ciliated epithelial cells propels the mucus layer together with entrapped pathogens, allergens, and environmental particles toward the pharynx for elimination, constituting an active defense mechanism. Concurrently, TJs, as key intercellular junctional structures, are formed by transmembrane molecules such as claudins and occludin, together with cytoplasmic scaffold proteins including zonula occludens-1/2/3 (ZO-1/2/3), assembling into dynamic complexes that regulate epithelial permeability. This restricts paracellular penetration of macromolecules and pathogens, thereby reinforcing barrier integrity (22).

**Table 1:** Overview of characteristic features of respiratory epithelial cell types.

Cell type	Morphological characteristics	Characteristic molecular markers	Differentiation and regulation	Immunoregulatory functions	Reference
Ciliated Cells (MCCs)	Columnar; 200–300 motile cilia (5–7 μm in length, “9 + 2” microtubule structure) at apical surface; abundant basal mitochondria; form tight junctions	Core markers: FOXJ1, RFX2, RFX3, MYB, TP73; Functional markers: AcTub, CCNO, DEUP1	Differentiated from basal cells; inhibited by Notch signaling; differentiation pathway: from basal cells via deuterosomal cells (expressing CDC20B, DEUP1) to mature MCCs; plastic, secretory cells may transdifferentiate under Notch inhibition	Ciliary beating drives mucociliary clearance; express TLR3/RIG-I to recognize viral PAMPs and secrete IFN-α/β; mediate Ca²^+^ signaling via connexin 43 gap junctions to synergize with macrophages in suppressing inflammation	(33–38)
Goblet Cells	Goblet-shaped; apical region filled with mucin granules (1–3 μm diameter); basal nucleus; form tight junctions with ciliated and basal cells	Mucin markers: MUC5AC (induced by inflammation), MUC5B (constitutive); Transcriptional regulators: SPDEF, FOXM1, RUNX2; Inhibitors: FOXA2, TTF-1	Differentiated from basal cells via parabasal intermediate; dependent on Notch1/2 activation; under homeostasis, FOXA2/TTF-1 suppress SPDEF; inflammation (e.g., IL-13) activates SPDEF and promotes proliferation	Secrete mucins containing antimicrobial peptides to entrap pathogens and toxins; express TLR4/TLR5, recognize LPS/flagellin to secrete IL-8 and TNF-α recruiting neutrophils; secrete TGF-β to suppress excessive inflammation	(33, 39–41)
Club Cells	Columnar or cuboidal; abundant apical smooth ER; contain Club cell secretory granules (0.5–1 μm, containing CCSP); central nucleus	Core markers: SCGB1A1 (CCSP), SCGB3A2; Functional markers: CYP450 enzymes, FOXA2, TTF-1	Primarily derived from “hillock basal cells” (TP63^+^/KRT13^+^); self-renew at steady state; can transdifferentiate into ciliated cells upon injury; Notch3 promotes differentiation; FOXA2/TTF1 maintain identity and inhibit goblet cell transdifferentiation	Detoxify xenobiotics via CYP450 enzymes; secrete CCSP to inhibit NF-κB pathway and reduce IL-8/TNF-α release; secrete PGE_2_ to promote M2 macrophage polarization; inhibit macrophage activation via CD200–CD200R interaction	(33, 34)
Basal Cells (BCs)	Located at basement membrane; cuboidal/polygonal; attached via hemidesmosomes; contain KRT5/KRT14; large, round nucleus with loose chromatin	Core markers: TP63, KRT5, KRT14, KRT15; Subpopulations: hillock basal cells (TP63^+^/KRT13^+^), proliferative basal cells (Ki67^+^)	Self-renew via p53/MDM2 pathway; ~40% annual turnover regulated by GM-CSF/mTORC1; multipotent: Notch inhibition → MCCs; Notch1/2 activation → secretory cells; Notch3 activation → parabasal cells; proliferate and differentiate into secretory cells upon injury, later into MCCs	Express TLR2/TLR4; secrete GM-CSF to recruit and activate macrophages; secrete TGF-β to suppress excessive T cell activation; after injury, secrete IL-6 to activate STAT3 pathway promoting proliferation/differentiation and tight junction expression	(33, 42, 43)
Pulmonary Neuroendocrine Cells (PNECs)	Round or columnar; contain dense-core neurosecretory granules (100–300 nm); often cluster as neuroepithelial bodies	CHGA, CGRP, SYP	Derived from basal cells; some subtypes self-renew; can transdifferentiate into secretory or ciliated cells after injury	Sense hypoxia or mechanical stimuli and secrete CGRP/Substance P to modulate vasodilation and immune cell activation; secrete IL-33 to activate ILC2s and regulate inflammation	(44–48)
Ionocytes	Short columnar; apical ion channels; mitochondria-rich; rare (0.5%–1% of airway epithelial cells)	FOXJ1, CFTR, TRPM5	Derived from basal or tuft cells; Notch signaling involved (mechanism not fully elucidated)	Regulate chloride transport via CFTR to maintain airway surface liquid homeostasis and mucus fluidity; secrete antimicrobial peptides e.g., β-defensins to inhibit pathogens	(49–51)
Tuft Cells	Columnar; apical tuft of microvilli; rich in ER and Golgi; rare (0.1%–0.5%)	POU2F3, TRPM5, IL-25	Derived from basal cells; potential promotion by Notch inhibition; plastic, can transdifferentiate into ionocytes or neuroendocrine cells	Upon recognition of pathogen metabolites, secrete IL-25 and TSLP to activate ILC2 and Th2 cells initiating type 2 immunity; express TLR9, recognize bacterial DNA to secrete TNF-α recruiting immune cells	(52, 53)

The stability of epithelial barriers is governed by multidimensional signaling networks, with exogenous and endogenous factors disrupting barrier integrity through distinct pathways. Viral proteins may directly interfere with epithelial polarity signaling (e.g., PALS1) or alter TJ protein expression (23). Reactive oxygen species (ROS), and metabolites such as ceramide further modulate TJ assembly and stability via TLR4-mediated integration of environmental signals (24, 25). Although direct evidence for IAV components disrupting TJ assembly remains lacking, indirect barrier injury mediated by cytokines and stress responses is well established. Moreover, viral PAMPs and ROS activate the TLR4–NF-κB pathway, driving the release of pro-inflammatory mediators such as TNF-α and IL-1β, thereby exacerbating epithelial barrier dysfunction. TNF-α markedly downregulates claudin-4/5, ZO-1, and β-catenin expression, induces actin cytoskeletal remodeling with stress fiber formation, and disrupts alveolar epithelial stability through ceramide accumulation, effects partially reversible by anti–TNF-α interventions (26). IL-1β enhances permeability via activation of the Transforming Growth Factor-β(TGF-β)/RhoA/αvβ6 integrin pathway (27). In addition, NF-κB cross-activates the Mitogen-Activated Protein Kinase (MAPK)/Phosphatidylinositol 3-Kinase (PI3K)–Gli1 pathway, upregulating the transcriptional repressor Snail, which suppresses E-cadherin, occludin, and ZO-1 transcription, ultimately disrupting TJ integrity, elevating permeability, and contributing to pulmonary edema and inflammatory injury (28).

Additionally, respiratory epithelial cells detect microenvironmental changes through pattern recognition receptors (PRRs) to coordinate innate immune responses. Toll-like receptors (TLRs) and retinoic acid–inducible gene I-like receptors (RLRs) recognize pathogen-associated molecular patterns (PAMPs). During IAV replication, Z-form RNA (Z-RNA) can be specifically recognized by Z-DNA-binding protein 1 (ZBP1) via its Zα domain. Activated ZBP1 initiates receptor-interacting protein kinase 3 (RIPK3)/mixed lineage kinase domain-like protein (MLKL)–dependent necroptosis, leading to nuclear membrane rupture and release of damage-associated molecular patterns (DAMPs) such as HMGB1 and nuclear DNA. These DAMPs recruit neutrophils and induce NETosis, amplifying inflammatory cascades (29).In addition, goblet and basal cells secrete epithelial “alarmins,” including interleukin-25 (IL-25) and thymic stromal lymphopoietin (TSLP), which activate type 2 innate lymphoid cells (ILC2s) and T helper 2 (Th2) responses (30). Single-cell transcriptomic studies further reveal that subsets of basal cells develop long-lasting epigenetic memory under interleukin-4 (IL-4)/interleukin-13 (IL-13) stimulation, sustaining pro-inflammatory cytokine secretion (31). In murine models, MLKL deficiency significantly reduces epithelial nuclear damage and neutrophil over-infiltration, improving survival after infection, highlighting epithelial cells as both structural barriers and central regulators of immune homeostasis (32).

In summary, the respiratory epithelium establishes a primary defense barrier through the mucus–ciliary system and TJs, while coordinating immune responses via PRRs, DAMP signaling, and cytokine networks. During IAV infection, ZBP1-mediated necroptosis and inflammatory mediator release disrupt epithelial homeostasis, and inflammatory and viral signaling pathways further exacerbate TJ impairment. Collectively, these mechanisms reveal the dual role of epithelial cells in antiviral defense and inflammatory pathology, providing mechanistic insights into respiratory infectious disease progression and potential therapeutic targets.

## PCD triggered by IAV: a double-edged sword in disease progression and immune regulation

**Figure 2:**
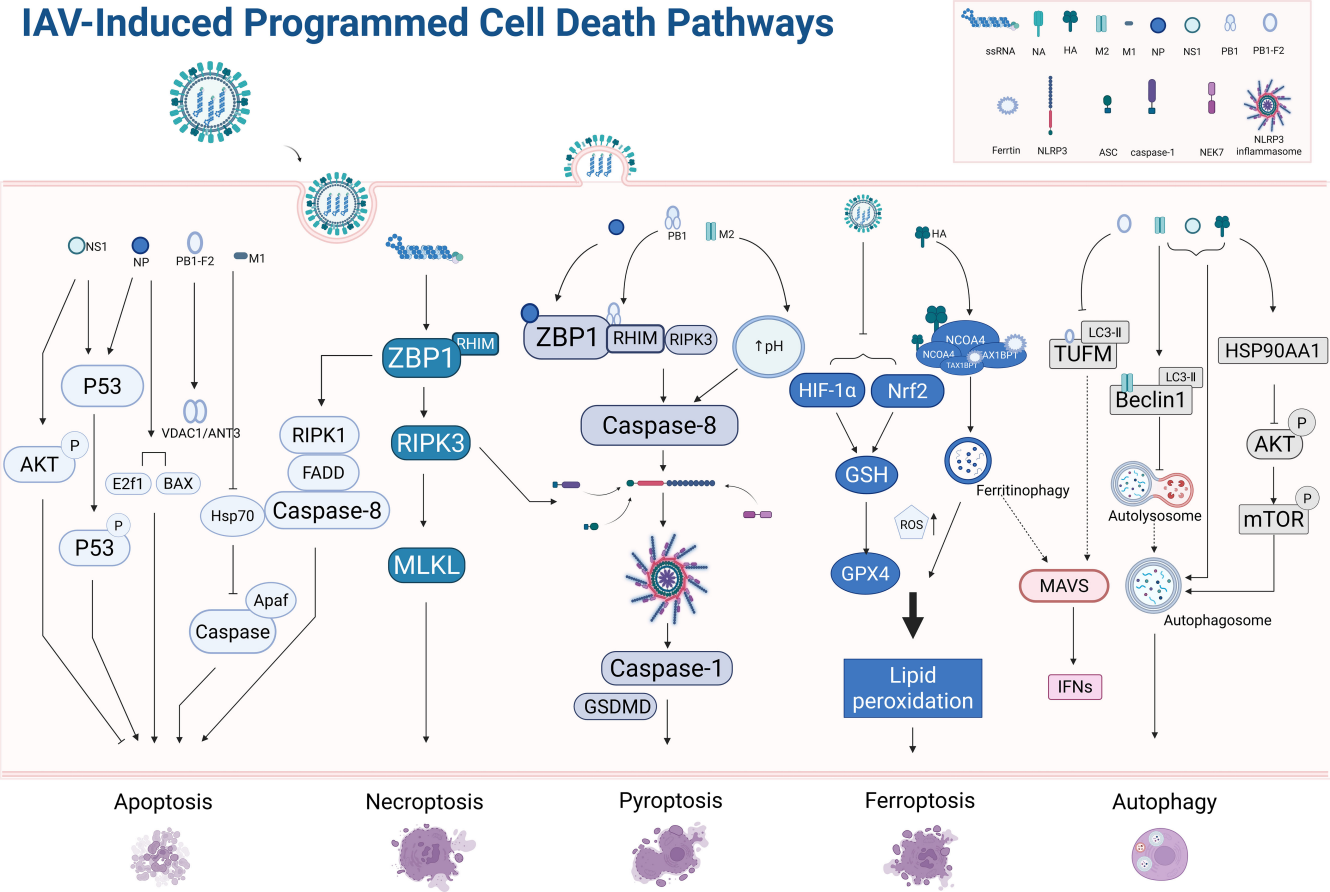
Schematic illustration of the distinct PCD pathways triggered by IAV infection: specific viral components, including structural proteins and replication intermediates, can activate distinct PCD modalities such as apoptosis, pyroptosis, necroptosis, and ferroptosis. Importantly, these pathways are not isolated; rather, they intersect through shared signaling mediators and regulatory nodes, shaping the balance between antiviral defense and immunopathology. Elucidating how IAV factors orchestrate the crosstalk among PCD pathways will provide critical insights into mechanisms of viral restriction, as well as strategies to alleviate excessive inflammation and tissue injury during infection. Created in BioRender. Luo, C. (2026) https://BioRender.com/s1pb7sj.

Influenza A virus (IAV) infection induces diverse forms of programmed cell death (PCD), including apoptosis, necroptosis, pyroptosis, ferroptosis, and PANoptosis. The underlying mechanism/key pathway is illustrated in **Figure 2**. These PCD pathways are differentially activated at distinct stages of the viral life cycle, profoundly shaping epithelial homeostasis and disease outcomes such as tissue injury and cytokine storm through modulation of immune responses and inflammation. Elucidating their activation mechanisms and regulatory crosstalk is central to understanding IAV pathogenesis and developing therapeutic strategies.

### Apoptosis: temporally orchestrated non-inflammatory cell clearance during IAV infection

During IAV infection, apoptosis undergoes a dynamic shift from anti-apoptotic to pro-apoptotic states, serving both viral replication and host defense. In the early stage, the viral nonstructural protein 1 (NS1) inhibits type I interferon production and activates the PI3K-AKT (Protein Kinase B, PKB) pathway to suppress apoptosis (54). NS1 also hijacks endoplasmic reticulum (ER) function by engaging the ER chaperone ERp57 to ensure proper HA folding and induce specific ER stress (e.g., ATF6/ERp57 upregulation), thereby creating a favorable environment for viral genome replication and assembly (55, 56).

At later stages, pro-apoptotic pathways are activated in a mitochondria-centered manner. The nucleoprotein (NP) stabilizes p53, derepresses BAX, and releases E2F1 to trigger intrinsic apoptosis (57–59). PB1-F2 disrupts mitochondrial membrane potential via Voltage-Dependent Anion Channel 1 (VDAC1) and Adenine Nucleotide Translocator 3 (ANT3) and amplifies extrinsic death receptor signaling (60). M1 relieves Heat Shock Protein 70 (HSP70)-mediated inhibition of Apoptotic Protease-Activating Factor 1 (APAF-1), facilitating caspase cascades (61). In parallel, IRF3, activates the Receptor-Interacting Protein Kinase 1 (RIPK1)-IFNβ activator (RIPA) pathway to induce mitochondrial apoptosis, though temporally suppressed by X-Linked Inhibitor of Apoptosis Protein (XIAP) to prevent premature cell death (62). Sustained ER stress further triggers caspase-12–dependent apoptosis independent of Fas and promotes TGF-β release via c-Jun N-Terminal Kinase 1 (JNK1) (63), potentially aiding tissue repair.

Collectively, IAV orchestrates apoptosis in a spatiotemporally regulated manner: delaying cell death early to favor replication and inducing apoptosis later to facilitate viral release. This non-inflammatory form of PCD supports viral spread and immune evasion while avoiding premature host clearance, with key molecular nodes (e.g., ERp57, NP-p53 axis, PB1-F2–mitochondrial pathway) representing potential antiviral targets.

### Autophagy: a regulatory hub balancing cytoprotection, inflammatory cell death and immune evasion

IAV manipulates autophagy at multiple levels, from initiation and maturation to selective pathways and immune interactions, establishing a complex network that facilitates efficient infection and pathogenicity.

During initiation, IAV suppresses the Mammalian Target of Rapamycin (mTOR) pathway to promote autophagy (64–66), with HA and NS1 cooperating to induce autophagosome formation, while later activation of PI3K–AKT or disruption of Microtubule-Associated Protein 1 Light Chain 3 (LC3)–Rab Guanosine Triphosphatase 11a (Rab11a) colocalization balances degradation. In maturation, M2 competitively binds Beclin 1 (BECN1), Autophagy-Related Gene 5 (ATG5), and LC3B (a subtype of LC3) to block autophagosome–lysosome fusion (67), leading to autophagosome accumulation that supports viral assembly and modulates downstream mTOR signaling.

In selective autophagy, PB1-F2 and NP promote MAVS degradation via mitophagy, weakening innate immunity (68–70); HA induces ferritinophagy to release iron, drive lipid peroxidation, and inhibit antiviral signaling (71); and noncanonical autophagy mediated by non-lipidated LC3 and Pericentrin (PCNT) facilitates vRNP uncoating (72).

At the immune level, IAV-induced autophagy enhances Major Histocompatibility Complex Class II (MHC II) antigen presentation via LC3-associated phagocytosis (LAP), while M2 suppresses Major Histocompatibility Complex Class I (MHC I) presentation (73). Mitophagy limits NLRP3 activation to prevent excessive inflammation (74, 75), ferritinophagy-derived lipid peroxidation inhibits interferon signaling (71), and degradation of Superoxide Dismutase 1 (SOD1) promotes ROS accumulation (76), collectively forming a positive feedback loop of “autophagy induction–immune suppression–replication enhancement.”

### Necroptosis: ZBP1–RIPK3–dependent inflammatory necrotic death in IAV infection

Necroptosis exerts dual roles in antiviral defense and immunopathology. It is initiated when ZBP1 senses viral Z-RNA generated during replication (77). Through its RHIM domain, ZBP1 interacts with RIPK3 (78), leading to (i) phosphorylation and oligomerization of MLKL, which disrupts the plasma membrane, and (ii) recruitment of the FADD–caspase-8 complex, linking necroptosis to apoptosis. Apoptosis and necroptosis are generally mutually exclusive within a single infected cell, with caspase-8 inhibition favoring necroptosis, whereas MLKL deficiency shifts toward apoptosis (79). Even in the absence of apoptosis, necroptosis can independently restrict viral replication and enhance CD8^+^ T cell responses, sometimes more effectively than apoptosis (80).

Functionally, necroptosis eliminates infected cells, releases DAMPs and viral antigens, activates dendritic cells, and promotes cross-presentation to boost adaptive immunity. However, excessive activation by highly pathogenic strains induces massive epithelial loss, barrier breakdown, edema, impaired gas exchange, and neutrophil over-recruitment, culminating in acute lung injury (ALI) and acute respiratory distress syndrome (ARDS) (32, 74, 81, 82).

Necroptosis outcomes are modulated by cIAP2-mediated ubiquitination and degradation of RIPK1/RIPK3 (74), viral HA subtype-specific regulation, and cell-type context (83). For example, in non-regenerable neurons, RIPK3 predominantly triggers chemokine expression rather than necroptosis to avoid irreversible inflammation (84).

### Pyroptosis: gasdermin-mediated inflammasome-dependent lytic cell death

Pyroptosis, an inflammatory form of PCD, is executed by gasdermin proteins and involves both antiviral and pathogenic roles during IAV infection. GSDMD and GSDME are the major effectors. GSDMD is cleaved by caspase-1 via the NOD-, LRR-, and Pyrin Domain-Containing Protein 3 (NLRP3) inflammasome (canonical pathway) or by caspase-4/5/11 (noncanonical pathway), generating N-terminal fragments that form plasma membrane pores, releasing IL-1β and IL-18 (78, 85–87). Gasdermin E (GSDME) is activated by caspase-3 or granzyme B, mediating apoptosis-to-pyroptosis switching, particularly during H7N9 infection, thereby exacerbating lung injury (88).

IAV proteins differentially regulate pyroptosis. M2 alters intracellular pH to activate NLRP3 (89), while PB1-F2 (H7N9/PR8) promotes ASC speck formation and inflammasome activation (90); however, PB1-F2 variants in H7N9 can suppress inflammasome assembly by disrupting MAVS or NIMA-Related Kinase 7 (NEK7) interactions (91). NS1 also suppresses NLRP3/ASC speck formation (92). On the host side, ZBP1 recognizes vRNP or Z-RNA and recruits RIPK3 and caspase-8 via RHIM, directly cleaving GSDMD or indirectly activating NLRP3 (93, 94). Its expression is interferon-inducible. Necroptosis-associated K^+^ efflux (95), as well as host proteins such as MxA and galectin-3 (96), also promote NLRP3 activation. Moderate pyroptosis aids viral clearance and immune activation, but excessive activation damages airway epithelia and drives cytokine storm, worsening pneumonia and ARDS.

### PANoptosis: ZBP1-driven integration of apoptotic, necroptotic and pyroptotic pathways

PANoptosis integrates apoptosis, necroptosis, and pyroptosis through assembly of the PANoptosome complex (77). ZBP1 recognizes IAV-derived Z-RNA and recruits RIPK3 via its RHIM domain, which in turn engages RIPK1 and caspase-8. This orchestrates MLKL-mediated necroptosis, caspase-3/7–driven apoptosis, and NLRP3-ASC–caspase-1–mediated GSDMD cleavage leading to pyroptosis. Such integration ensures redundant clearance of infected cells even if one pathway is blocked (e.g., caspase-8, RIPK3, or MLKL inhibition). Biologically, PANoptosis enhances immune activation through DAMP and cytokine release (82), promoting cell recruitment and antigen cross-presentation, but its overactivation exacerbates tissue injury and cytokine storm, driving ALI.

### Ferroptosis: iron-dependent oxidative cell death driven by lipid peroxidation during IAV infection

Ferroptosis is characterized by iron-dependent lipid peroxidation and contributes to epithelial damage during IAV infection.

Viral modulation of iron metabolism includes upregulation of Transferrin Receptor 1 (TfR1) to enhance iron uptake, suppression of Ferroportin 1 (FPN1) to block iron export, and induction of ferritinophagy via Nuclear Receptor Coactivator 4 (NCOA4), releasing labile iron. HA interactions with NCOA4/TAX1-Binding Protein 1 (TAX1BP1) accelerate ferritin degradation, expanding the labile iron pool and driving ROS production through Fenton chemistry and Lipoxygenase (LOX)-mediated Polyunsaturated Fatty Acid (PUFA) oxidation (71).

IAV disrupts antioxidant defenses by inhibiting Solute Carrier Family 7 Member 11 (SLC7A11) and Glutathione Peroxidase 4 (GPX4), depleting Glutathione (GSH) (97), and targeting Nuclear Factor Erythroid 2-Related Factor 2 (NRF2) through Interferon-Inducible Protein 35 (IFP35), Tripartite Motif Protein 21 (TRIM21), and Tripartite Motif Protein 46 (TRIM46)-mediated pathways (98). H1N1 further suppresses the NRF2–Glutamate-Cysteine Ligase Catalytic Subunit (GCLC) axis, impairing GSH synthesis.

Concurrently, mitochondrial dysfunction (via Acyl-CoA Synthetase 4 (ACSL4) upregulation, PB1-F2/M2/NS1-mediated disruption, and Dihydroorotate Dehydrogenase (DHODH) inhibition) (99), peroxisome (PX) degradation (pexophagy), and Indoleamine 2,3-Dioxygenase 1 (IDO1)–Inducible Nitric Oxide Synthase (iNOS)–mediated ONOO^-^ production collectively intensify lipid peroxidation (100).

In summary, during IAV infection, multiple forms of PCD-including apoptosis, necroptosis, pyroptosis, PANoptosis, ferroptosis, and autophagy-associated cell death-do not operate as independent linear pathways but instead constitute a highly dynamic and hierarchically integrated regulatory network. These death modalities exhibit substantial overlap at the levels of upstream sensing molecules, signaling intermediates, and execution mechanisms, resulting in extensive crosstalk, functional compensation, and context-dependent dominance. The apparent mutual exclusivity observed between pathways such as apoptosis and necroptosis primarily arises from competitive regulation at key molecular nodes rather than from fixed pathway insulation.

Core molecules including ZBP1, RIPK1, RIPK3, and caspase-8 function as integrative hubs that couple viral nucleic acid recognition to downstream cell death execution programs. Recognition of IAV replication–derived Z-RNA by ZBP1 promotes the assembly of multiprotein signaling complexes, enabling the concurrent initiation of apoptotic, necroptotic, and pyroptotic signaling. Caspase-8 serves as a critical molecular switch within this network: its activation preferentially drives apoptosis while suppressing RIPK3–MLKL–mediated necroptosis, whereas functional inhibition or depletion of caspase-8 permits necroptosis or PANoptosis to become dominant. Similarly, gasdermin cleavage mediated by caspase-1 or caspase-3 can redirect canonical apoptotic signaling toward pyroptotic execution, highlighting the pronounced plasticity of PCD execution at the terminal stage.

The temporal progression of infection represents a key determinant of PCD hierarchy and inflammatory properties. During the early phase of infection, IAV preferentially induces non-lytic or weakly inflammatory processes, such as apoptosis and autophagy-associated pathways, thereby supporting viral replication and assembly while limiting premature inflammatory responses. As viral replication intensifies and viral RNA, viral proteins, and cellular stress signals accumulate, inflammatory PCD modalities-including necroptosis, pyroptosis, and PANoptosis-gradually predominate, driven by amplified interferon signaling, inflammasome activation, and mitochondrial dysfunction. Ferroptosis intersects with these inflammatory pathways under conditions of sustained oxidative stress and iron dysregulation, further exacerbating epithelial damage during advanced stages of disease.

The selection and execution of specific PCD outcomes are further shaped by the combined influence of viral determinants, host regulatory networks, and intrinsic properties of infected cells. Viral proteins such as NS1, PB1-F2, M2, and HA concurrently modulate multiple PCD pathways, while host factors-including ubiquitin ligases, antioxidant systems, interferon-stimulated genes, and autophagic machinery-fine-tune activation thresholds and execution efficiency. Moreover, cell type–specific differences in regenerative capacity, metabolic state, and expression of death-regulatory factors impose additional constraints, resulting in distinct PCD outcomes across different cellular contexts. Together, these multilayered regulatory mechanisms define the spatiotemporal pattern of PCD during IAV infection and profoundly influence immune activation, tissue injury, and disease severity.

## PCD-mediated immune microenvironment remodeling in IAV infection

### Interplay of PRRs, inflammasomes, and PCD in antiviral defense

During Influenza A Virus (IAV) infection, respiratory epithelial cells detect viral pathogen-associated molecular patterns (PAMPs) through pattern recognition receptors (PRRs), thereby initiating antiviral defense and inflammatory responses (101). Following HA-mediated entry, vRNPs are released into the cytoplasm and translocated into the nucleus for replication, while viral RNAs and associated components are recognized by multiple PRRs. Endosomal TLR3 senses double-stranded RNA (dsRNA) and activates IRF3 via TIR Domain-Containing Adaptor Protein Inducing IFN-β (TRIF), inducing IFN-β production. TLR7 detects single-stranded RNA (ssRNA) and triggers IRF7 and NF-κB activation through a Myeloid Differentiation Primary Response 88 (MyD88)-dependent pathway, thereby promoting IFN-α and proinflammatory cytokine transcription. TLR10 may also contribute to viral recognition, although its mechanism remains unclear (102). In the cytoplasm, RIG-I acts as a pivotal sensor of 5′-triphosphate RNA (5′pppRNA), undergoing TRIM25-mediated ubiquitination and activating the MAVS signaling platform, which drives TANK-Binding Kinase 1 (TBK1)/IκB Kinase ϵ (IKKϵ), IRF3/7, and NF-κB pathways, generating a potent interferon response (103)(Regarding the various types of interferons, relevant information is provided in **Table 2**). Secreted type I interferons subsequently engage the Janus Kinase-Signal Transducer and Activator of Transcription (JAK-STAT) pathway to induce interferon-stimulated genes (ISGs) (104), establishing a broad-spectrum antiviral state that intersects with programmed cell death (PCD) signaling to regulate inflammatory responses.

**Table 2:** Overview of interferon classification and their biological characteristics.

Type	Members	Receptor complex	Signal transduction pathway	Major cellular sources	Functional characteristics	Reference
Type I IFN	IFN-α (multiple subtypes in humans), IFN-β; minor: IFN-ϵ, κ, ω, ζ (mouse), τ (ruminants)	IFNAR1/IFNAR2	JAK1/TYK2 → STAT1/STAT2 + IRF9 → ISGF3 → ISRE → ISGs; partial activation of STAT1 homodimer → GAS	Almost all cell types (epithelial, fibroblasts, etc.); pDCs are professional high producers	Broad-spectrum and rapid antiviral activity; induction of ISGs (e.g., PKR, Mx); systemic effects; immunomodulation; excessive levels may cause systemic inflammation and tissue damage	(122–128)
Type II IFN	Only IFN-γ	IFNGR1/IFNGR2	JAK1/JAK2 → STAT1 homodimer (GAF) → GAS → ISGs; weak activation of ISGF3	Activated T cells (Th1, CTL), NK cells; minor: B cells, DCs	Primarily immunomodulatory (promotes Th1 responses, macrophage activation, antigen presentation); defense against intracellular pathogens; acts later in infection; synergy with TNF-α may cause excessive inflammation and cell death	(126, 129, 130)
Type III IFN	Human: IFN-λ1/λ2/λ3 (IL-29/28A/28B), IFN-λ4 (non-functional in some populations); Mouse: IFN-λ2/λ3	IFNLR1/IL-10R2	JAK1/TYK2 → STAT1/STAT2 + IRF9 → ISGF3 → ISRE → ISGs (same as Type I but slower and more sustained)	Mucosal epithelial cells (e.g., respiratory, intestinal tracts); pDCs, macrophages (auxiliary secretion)	Local mucosal antiviral defense; low systemic inflammation; promotes epithelial repair; dependent on NF-κB and IRF1 (independent of AP-1); associated with milder disease symptoms	(123, 131–134)

Simultaneously, NOD-like receptors (NLRs) contribute critically to immune microenvironment remodeling via inflammasome activation. NLRP3 can be activated by multiple IAV-derived stimuli, including viral RNA, M2 ion channel–mediated proton flux, and PB1-F2 protein aggregation (92, 95, 105, 106). NLRP3 inflammasome assembly promotes caspase-1–mediated cleavage of IL-1β and IL-18, amplifying inflammation and reinforcing interferon signaling (107). Absent in Melanoma 2 (AIM2) inflammasomes can be triggered by recognition of double-stranded DNA (dsDNA) or oxidized DNA (108), while the interferon-inducible protein Myxovirus Resistance Protein A (MxA) may interact with Apoptosis-Associated Speck-Like Protein Containing a CARD (ASC) to participate in inflammasome responses (109), although certain viral strains can evade detection. Inflammasome activation not only drives pyroptosis but also regulates immune cell recruitment and differentiation through cytokine release, shaping the infection milieu. Moreover, RIPK3 phosphorylates MLKL to mediate potassium efflux, facilitating NLRP3 activation, IL-1β maturation, and necroptosis (78). In the absence of functional MLKL, caspase-8 can process IL-1β and induce apoptosis via the ZBP1–RIPK3 complex directly or through caspase-3 (79), demonstrating redundancy between necroptotic and apoptotic pathways. Double knockout of MLKL and caspase-8 nearly abolishes IL-1β release and delays cell death, highlighting a multi-layered inflammasome–cell death axis that ensures sustained inflammatory signaling.

Interferon and PCD pathways are tightly interconnected through bidirectional regulation. Interferons can upregulate Tumor Necrosis Factor-Related Apoptosis-Inducing Ligand (TRAIL) expression, which engages Death Receptor 5 (DR5) on target cells to activate caspase-8/10 signaling, thereby promoting apoptosis of infected cells and restricting viral dissemination (110). TRAIL secretion by CD8^+^ T cells and macrophages further enhances immune clearance. However, excessive TRAIL activation may induce apoptosis of uninfected epithelial cells and impair alveolar fluid clearance by Adenosine Monophosphate-Activated Protein Kinase (AMPK)-mediated suppression of Na^+^,K^+^-ATPase, leading to pulmonary edema, particularly in highly pathogenic strains (111). Interferons can also drive ZBP1-dependent necroptosis and PANoptosis (112, 113), and regulate cell fate through p53-dependent induction of Growth Arrest and DNA Damage-Inducible Protein 45γ (Gadd45g) and Dual-Specificity Phosphatase 5 (Dusp5), which modulate cell cycle and promote apoptosis (114, 115), thus coupling viral clearance with epithelial repair dynamics (116). Conversely, hyperactive interferon responses—especially interferon-λ (IFN-λ)—can suppress alveolar epithelial proliferation and differentiation, delay tissue regeneration, and increase susceptibility to secondary bacterial infections (117).

To prevent immunopathology, the host relies on multiple negative regulatory mechanisms to fine-tune interferon signaling. Viral NS1 inhibits RIG-I and Protein Kinase R (PKR) activation (118, 119), PB1-F2 and PB2 promote MAVS degradation via autophagy (10, 120), Suppressor of Cytokine Signaling 1 (SOCS1) suppresses the JAK-STAT cascade, and the long non-coding RNA (lncRNA) Influenza Virus-Associated Long Non-Coding RNA (NRAV) represses ISG transcription (121). Together, these self-limiting processes restrain excessive inflammation and immune-mediated damage.

In summary, during IAV infection, PRRs detect viral RNA to initiate interferon responses and ISG induction, suppressing viral replication while driving PCD through TRAIL and ZBP1-dependent pathways to eliminate infected cells. Inflammasome–PCD crosstalk further regulates immune cell recruitment and differentiation, shaping the local immune microenvironment. Moderate activation promotes antiviral defense, whereas excessive or sustained activation causes epithelial injury and impaired repair. Ultimately, host-derived negative feedback ensures dynamic equilibrium between interferon signaling and PCD, balancing antiviral efficacy with immune pathology and influencing infection outcomes.

### The Role of the cell death–inflammation axis in immune cell recruitment and functional polarization

**Figure 3:**
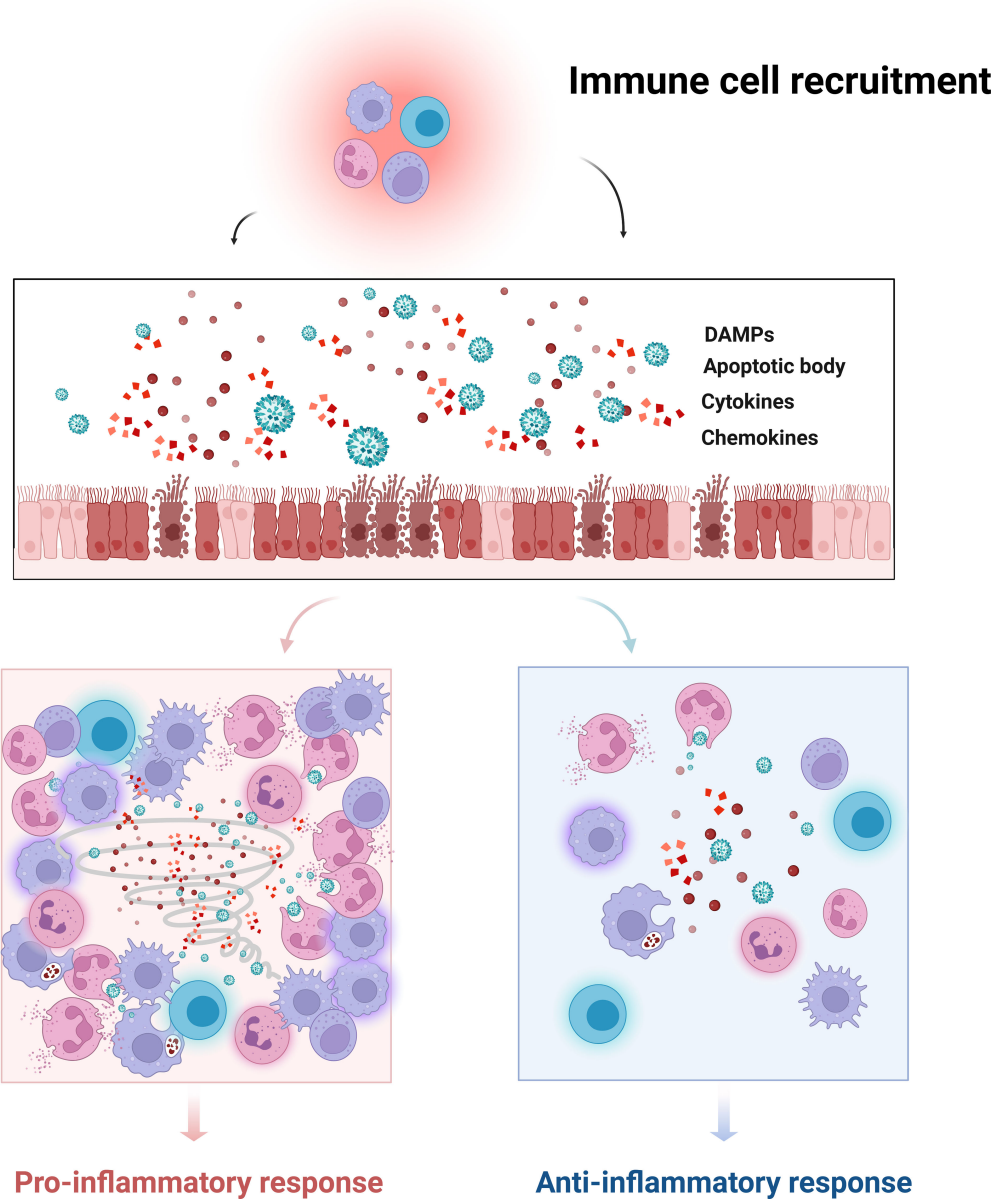
Crosstalk between IAV-induced PCD and immune responses: in the context of IAV infection, PCD of pulmonary epithelial cells represents a pivotal event linking viral clearance and immune regulation. On the one hand, epithelial PCD facilitates the removal of infected cells but is accompanied by the release of DAMPs, proinflammatory cytokines, and chemokines. These mediators orchestrate the recruitment and activation of diverse immune cells, including macrophages, neutrophils, and lymphocytes. Moderate levels of immune cell influx and activation are beneficial, as they enhance antiviral defense and restrict viral replication. However, when PCD-driven signals lead to excessive immune cell recruitment and hyperactivation, the process becomes detrimental. Overactivation and even death of infiltrating immune cells amplify inflammatory signaling, triggering a self-reinforcing cascade that culminates in cytokine storm. This exaggerated inflammatory response not only aggravates epithelial and vascular injury but also drives uncontrolled tissue damage, edema, and clinical deterioration. Created in BioRender. Luo, C. (2026) https://BioRender.com/y2kwrph.

During IAV infection, infiltrating inflammatory immune cells arise as a consequence of both virus- and host-derived signals activating canonical immune pathways and constitute a major pathological component operating in parallel with epithelial cell death (135). The complete workflow of this process is shown in **Figure 3**. Viral PAMPs together with DAMPs released from dying cells cooperatively initiate innate immune responses (107). Pattern recognition receptors such as RIG-I and TLRs activate type I interferon signaling, NF-κB pathways, and the NLRP3 inflammasome, thereby promoting the production of pro-inflammatory mediators including IL-1β and TNF-α (107, 136). These mediators drive the recruitment of monocytes and neutrophils to the lung and activate resident macrophages and dendritic cells. Subsequently, antigen-presenting cells migrate to draining lymph nodes to initiate virus-specific CD8^+^ and CD4^+^ T cell responses (137, 138). Activated T cells, together with persistently released DAMPs, further amplify immune cell infiltration and tissue inflammation, forming a self-reinforcing pathological loop. Importantly, the aberrant recruitment and functional polarization of immune cells during IAV infection are not governed by a single immune pathway but are profoundly shaped by the dynamic regulation of multiple PCD modalities throughout the course of infection.

Distinct forms of cell death differentially determine the spectrum and magnitude of inflammatory mediators and DAMPs released, while also selectively engaging inflammasomes, death-inducing signaling complexes, and antigen presentation pathways. Through these mechanisms, PCD finely tunes the intensity of immune cell recruitment, effector function, and cellular survival fate. Consequently, elucidating the roles of diverse PCD programs in immune cell regulation during IAV infection is essential for understanding how the “cell death–inflammation axis” evolves into immunopathological tissue damage.

Influenza A virus (IAV) infection induces multiple forms of programmed cell death (PCD), including pyroptosis, necroptosis, apoptosis, and NETosis. These distinct death pathways regulate immune cell recruitment, activation, and death through the release of inflammatory mediators and activation of signaling cascades, ultimately shaping infection outcomes.

Pyroptosis, an inflammatory lytic form of cell death, centers on inflammasome activation. NLRP3, NOD-Like Receptor Family, CARD Domain-Containing Protein 4 (NLRC4), and AIM2 inflammasomes activate caspase-1, which cleaves pro-IL-1β and pro-IL-18 into their mature forms (139, 140). IL-1β drives neutrophil recruitment, while IL-18 stimulates NK cells to produce interferon-γ (IFN-γ), thereby enhancing macrophage effector functions (132). During pyroptosis, pore-induced intracellular traps (PITs) sequester intracellular pathogens, expose complement-binding sites, and release lipid mediators to recruit neutrophils and macrophages for phagocytic clearance (141). Although pyroptosis contributes little to direct pathogen killing, it indirectly reinforces local antimicrobial defenses by activating immune cells through cytokine release and PITs. Excessive pyroptosis can result in uncontrolled cytokine release, exacerbating immune cell infiltration and tissue injury.

Necroptosis, mediated through the RIPK3–MLKL axis, is characterized by inflammatory lytic features. In IAV infection, ZBP1 senses viral RNA and activates RIPK3–MLKL signaling, leading to cell rupture and the release of damage-associated molecular patterns (DAMPs). These signals recruit NK cells and macrophages, amplifying inflammation. Necroptosis enhances immune cell infiltration and viral clearance but can also intensify pulmonary injury when inflammation is excessive. Its effects are pathogen-specific (16, 74): necroptosis correlates positively with immune infiltration during influenza infection but has limited impact in bacterial infections, highlighting pathogen-dependent immune regulation.

In contrast, apoptosis represents a non-inflammatory form of cell death critical for maintaining immune homeostasis. Apoptotic bodies are cleared by macrophages via ‘find-me’ and ‘eat-me’ signals, preventing excessive DAMP accumulation while enabling antigen cross-presentation to Cluster of Differentiation 8-Positive T Cells (CD8^+^ T Cells and strengthening adaptive immunity (142). Crosstalk between caspase-8 and RIPK3 provides a safeguard mechanism (143), ensuring that when one pathway is blocked, the other is engaged, thus maintaining the balance of immune cell activation and death and influencing disease prognosis.

NETosis, a neutrophil-specific death program, directly modulates immune responses through the release of NETs (18). Composed of chromatin and antimicrobial proteins, NETs capture and kill extracellular pathogens while amplifying inflammation in cooperation with platelets and macrophages. Platelets release chemokines to recruit additional neutrophils, whereas macrophages phagocytose NETs and their entrapped pathogens to promote clearance (144, 145). While NETs aid in pathogen control, excessive NETosis can drive immune cell exhaustion and tissue damage (146, 147).

At the lymphocyte level, IAV infection directly impacts T cell fate. H1N1 preferentially targets CD8^+^ effector memory T cells (T_em_ cells), which highly express α2,6-SA receptors, facilitating viral entry and triggering translational arrest and apoptotic signaling (148). In infected T cells, ribosomal proteins and translation factors are downregulated, proinflammatory mediators such as IL-1β are suppressed, while metabolic and antiviral genes (e.g., IDO1, IFITM3) are upregulated, collectively driving apoptosis. Apoptotic T cells release viral antigens that dendritic cells (DCs) can cross-present to activate cytotoxic T lymphocytes (CTLs), yet excessive T cell loss weakens adaptive immunity. Concurrently, activation of inhibitory pathways, including Programmed Death 1 (PD-1) and Lymphocyte Activation Gene 3 (LAG-3) (149, 150), promotes CD8^+^ T cell exhaustion, impairing long-term immune protection and underscoring the regulatory role of immune checkpoint pathways in disease outcome.

In macrophages, IAV activates the TLR4–NF-κB–Tumor Necrosis Factor (TNF) axis, which in turn engages RIPK1/RIPK3–MLKL signaling, inducing necroptosis and releasing abundant DAMPs and viral antigens. These signals are processed by dendritic cells, promoting antigen presentation and T cell activation. However, excessive activation drives persistent immune cell death and inflammatory imbalance. Caspase-6 acts as a scaffold to amplify ZBP1-dependent inflammasome activation and participates in PANoptosis (coordinated activation of pyroptosis, necroptosis, and apoptosis), thereby regulating macrophage polarization and the immune microenvironment.

In summary, IAV-induced PCD profoundly influences immune cell recruitment, function, and fate through cytokine release, antigen presentation, and signaling activation. Beyond epithelial cell death, excessive activation, effector amplification, and programmed death of immune cells themselves constitute major drivers of pulmonary tissue injury during influenza infection. Pyroptosis and necroptosis amplify inflammation and immune infiltration but can cause collateral tissue damage when excessive; apoptosis constrains inflammation while facilitating cross-presentation to sustain immune homeostasis; NETosis aids pathogen clearance but risks immune pathology; and T cell and macrophage death critically determine the strength and persistence of adaptive immune responses. Collectively, the synergy and dysregulation between diverse PCD pathways and canonical immune activation cascades define the immunopathological trajectory of influenza disease.

### Epithelial repair networks in host tolerance: crosstalk between cell death and immune signaling

Infection with influenza A virus (IAV) directly disrupts the respiratory epithelial barrier, leading to structural damage and functional impairment. To counter viral invasion, the host relies both on epithelial tolerance mechanisms that restrict damage propagation and on multilayered repair processes that restore barrier integrity. Notably, epithelial injury and immune dysregulation induced by IAV infection are not confined to the acute phase of disease. An increasing body of evidence indicates that, even after viral clearance, a subset of hosts develops persistent immunopathological alterations, characterized by chronic inflammation, aberrant tissue repair, and incomplete functional recovery. This post-acute immune dysregulation is closely associated with sustained epithelial damage, the residual consequences of programmed cell death, and impaired coordination of immune responses (135, 151). Collectively, these processes may establish a pathogenic foundation for long-term pulmonary dysfunction, a predisposition to fibrotic remodeling, and heightened susceptibility to secondary infections.

At the early stage of infection, ciliated cells, secretory cells, and type II alveolar epithelial cells (AT2 cells) collaborate to provide front-line defense (152). Ciliated cells eliminate virus-laden particles via the mucociliary clearance system, while secretory cells release large amounts of mucins (MUC5AC/MUC5B), whose sialylated residues act as decoy receptors binding viral hemagglutinin (HA) and limiting engagement with host receptors (153). Concurrently, epithelial cells secrete β-defensins and the antimicrobial peptide LL37, which disrupt viral membranes, whereas AT2 cells produce surfactant proteins A/D (SP-A/SP-D) to enhance macrophage phagocytosis (154). Collectively, these mechanisms form a multilayered barrier that restricts viral spread. If the virus overcomes these defenses, epithelial cells detect viral RNA through pattern recognition receptors (PRRs; TLR3/7, RIG-I, MDA5), activating NF-κB and IRF3/7 pathways to induce type I and type III interferon secretion. These, in turn, stimulate interferon-stimulated genes (ISGs; e.g., IFITM3, Mx, PKR) to establish an antiviral state. Meanwhile, NLRP3 inflammasome activation promotes maturation of IL-1β and IL-18, which, together with IL-6 and CCL2, recruit neutrophils, dendritic cells, and CD8^+^ T cells to eliminate infected cells, while TGF-β modulates the balance between inflammation and repair (155).

As infection progresses, epithelial barrier disruption triggers cell death and tissue injury. Apoptotic body clearance and Rac Guanosine Triphosphatase 1 (Rac1)-dependent epithelial autophagy remove necrotic debris, thereby limiting DAMP accumulation and secondary inflammation (156). During tissue remodeling, airway basal cells, acting as progenitor reserves, are activated to upregulate keratin 5 (KRT5) and keratin 17 (KRT17), proliferating and differentiating into ciliated and secretory cells to restore airway epithelium. Ciliated cell precursors gradually differentiate into mature ciliated cells through upregulation of genes such as Tubulin Beta-4B Chain (TUBB4B). In the alveolar region, AT2 cells serve as central repair mediators, proliferating under Wnt and fibroblast growth factor (FGF) signaling and differentiating into AT1 cells via β-catenin and Smad pathways to reestablish gas exchange (157). Bone Marrow-Derived Macrophages (BMDMs) secrete growth factors such as Insulin-Like Growth Factor 1 (IGF-1) to promote barrier reconstruction (158), while restoration of TJ proteins (ZO-1, Claudins) and epithelial sodium channels (ENaC) ensures alveolar fluid clearance and barrier repair (159).

Immune cells and key signaling molecules play central roles in tolerance and repair. BMDMs dynamically transition between inflammatory (BMDM1) and regenerative (BMDM2) states; the latter secrete pleiotrophin (Plet1), which promotes AT2 proliferation via Raf-Mitogen-Activated Protein Kinase Kinase (MEK)-Extracellular Signal-Regulated Kinase (ERK) signaling and enhances tight junction protein expression by inhibiting Src kinase activity, thereby restoring transepithelial resistance (160). Regulatory T Cells (Tregs) secrete amphiregulin (Areg), an Epidermal Growth Factor Receptor (EGFR) ligand, which acts on Collagen Type XIV Alpha 1 Chain (Col14a1)^+^ stromal cells to induce FGF7/10 production, indirectly regulating AT2 proliferation and differentiation. Tregs also facilitate the formation of transitional alveolar epithelial cells (AECint), enabling AT2-to-AT1 conversion (161). NK cells sustain IL-22 secretion through Cyclophilin D (CypD)-dependent metabolic regulation; IL-22 promotes epithelial proliferation and junctional integrity (162), whereas CypD deficiency reduces IL-22, exacerbating epithelial injury. Granulocyte-Macrophage Colony-Stimulating Factor (GM-CSF) and Areg further support epithelial regeneration and immune tolerance, while integrin αvβ6 regulates TGF-β and interferon signaling to maintain pulmonary homeostasis (163, 164).

Conversely, IAV infection can induce epithelial and macrophage senescence, leading to a senescence-associated secretory phenotype (SASP) that releases IL-1β and profibrotic factors (165), impeding epithelial regeneration and driving chronic lung damage. Even after viral clearance, senescence propagates to parenchymal cells via paracrine signaling, forming a “senescence–repair blockade” cycle, highlighting the importance of senescent cell clearance in promoting epithelial repair. In addition, vitamin D exerts multifaceted protective effects (166): its active form, 1,25-dihydroxyvitamin D_3_ [1,25(OH)_2_D_3_], promotes basal cell proliferation and tight junction expression via the Vitamin D Receptor (VDR)-Phosphatase and Tensin Homolog (PTEN) pathway, enhances transepithelial resistance, upregulates LL37 to restrict viral replication, and suppresses excessive IL-6 and IFN-β expression to prevent secondary immune-mediated damage.

In summary, IAV-induced epithelial barrier disruption activates a multilayered defense and repair network, spanning innate defense, immune regulation, and tissue regeneration. This process relies on the coordinated actions of key molecules and diverse immune cell subsets to maintain a dynamic balance between antiviral defense, inflammation tolerance, and epithelial repair. Such complexity not only underpins host survival and recovery but also provides potential targets for therapeutic intervention in virus-induced lung injury.

## Intervention strategies against IAV via targeting PCD pathways

**Figure 4:**
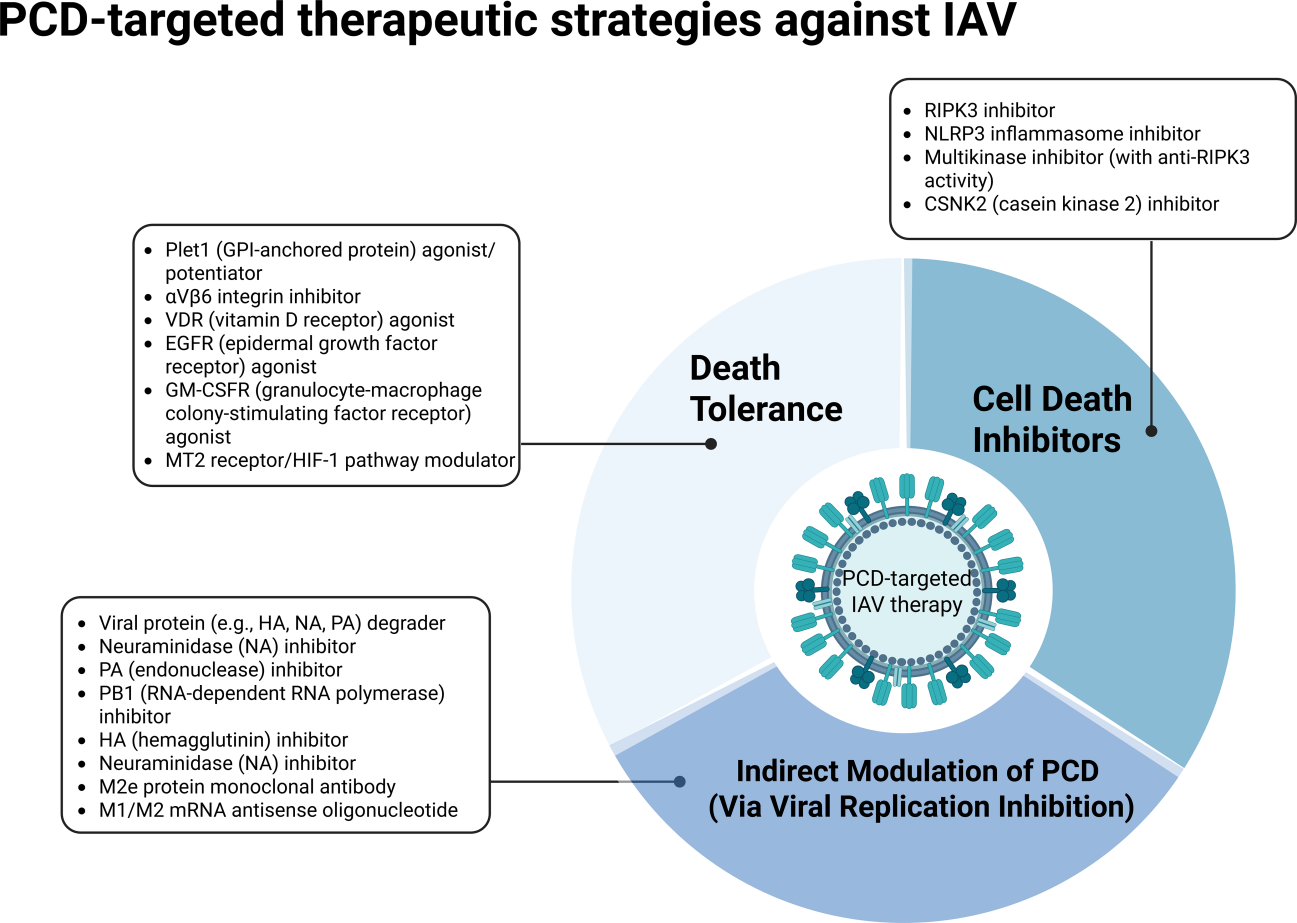
PCD-targeted therapeutic strategies against (IAV). Created in BioRender. Luo, C. (2026) https://BioRender.com/ep2f8rs.

Although multiple classes of antiviral agents-such as the neuraminidase inhibitor oseltamivir and the RNA polymerase inhibitor baloxavir-have been approved for clinical management of IAV infection, their therapeutic efficacy remains constrained by several factors, including the rapid emergence of drug-resistant viral strains, a narrow antiviral spectrum, and suboptimal outcomes in severe or late-stage infections (177, 187). These limitations have prompted increasing attention toward host-targeted therapeutic strategies, particularly interventions aimed at virus-induced PCD pathways (see the classification framework in **Figure 4** and the specific agents in **Table 3**). Extensive evidence indicates that IAV infection can activate diverse PCD modalities, including necroptosis and pyroptosis, which not only directly compromise the integrity of the respiratory epithelial barrier but also amplify inflammatory responses, thereby exacerbating disease progression. Consequently, pharmacological strategies that target key regulators of PCD have emerged as promising therapeutic avenues-for instance, inhibition of the ZBP1-RIPK3 signaling axis to block necroptosis (167), or suppression of the NLRP3 inflammasome to mitigate pyroptosis-associated hyperinflammation (74). Moreover, strategies that enhance host tolerance to PCD-reducing pathological cell death without directly inhibiting viral replication-have demonstrated protective effects in multiple preclinical models.

**Table 3:** Therapeutic Approaches for IAV by Targeting the Regulation of PCD Pathways: The table highlights three principal categories of interventions.

Name (generic or code)	Target	Mechanism summary	Development stage/approval status	Notes (spectrum, resistance, safety, etc.)	Reference
Cell death inhibitors					
UH15-38	RIPK3	Potently inhibits RIPK3 kinase activity, blocks MLKL phosphorylation, and specifically suppresses IAV-induced necroptosis.	Preclinical research	Effective against multiple IAV and IBV strains; well-tolerated *in vivo* with a wide therapeutic window.	(167, 168)
MCC950	NLRP3 Inflammasome	Specifically inhibits NLRP3 activation, reducing caspase-1 cleavage and IL-1β/IL-18 release to mitigate pyroptosis-driven inflammation. Does not impair antiviral IFN response or viral clearance.	Preclinical research	Protective in A/WSN/1933 and other NLRP3-related diseases; early use may impair immune responses; limited to NLRP3-mediated pyroptosis.	(163, 169)
Ponatinib	RIPK3, BCR-ABL, etc.	Multi-kinase inhibitor that unexpectedly inhibits RIPK3, thereby blocking necroptosis.	Approved (for leukemia)	Non-specific; significant side effects; not suitable for IAV treatment.	(170, 171)
CX-4945 (Silmitasertib)	CSNK2 (Casein kinase 2)	Inhibits CSNK2, enhances autophagy, and indirectly reduces epithelial damage associated with excessive PCD.	Phase II clinical trials (for cancer)	Potential broad-spectrum activity against RNA viruses; safety profile requires monitoring.	(172–174)
Death tolerance					
Recombinant Plet1 (rPlet1)	Plet1 (GPI-anchored protein)	Promotes proliferation of alveolar epithelial progenitor cells, enhances epithelial barrier integrity, and repairs IAV-induced PCD damage without directly affecting viral replication.	Preclinical research	Improves survival rates; favorable safety profile; wide therapeutic window.	(160)
αVβ6 Integrin Inhibitor (Antibody)	αVβ6 Integrin	Blocks TGF-β activation, enhances macrophage function, increases Type I interferon levels, and reduces inflammation and PCD-related damage.	Preclinical research	Host-targeted mechanism; no resistance concerns; suitable for short-term intervention.	(164)
1,25-Dihydroxyvitamin D3	VDR (Vitamin D receptor)	Upregulates antimicrobial peptides, inhibits inflammatory cytokines, promotes epithelial repair, and reduces PCD.	Approved (for other indications)	Requires optimized administration; long-term high doses may cause hypercalcemia.	(166)
Amphiregulin (AREG)	EGFR	Activates EGFR-ERK pathway, promotes epithelial repair, enhances anti-inflammatory responses, and indirectly reduces PCD.	Preclinical research	Should be administered during the repair phase; use during acute phase may risk fibrosis.	(174)
CypD Inhibitor (e.g., Cyclosporin A)	Cyclophilin D (CypD)	Inhibits mPTP opening, modulates NK cell function and IL-22 production, enhances disease tolerance, and indirectly regulates PCD.	Approved (for organ transplantation)	Long-term use carries risks such as nephrotoxicity; requires evaluation for IAV indication.	(162)
GM-CSF	GM-CSFR	Enhances macrophage function, promotes epithelial proliferation, modulates macrophage polarization, and indirectly reduces PCD.	Approved (for neutropenia)	High doses may cause capillary leak syndrome.	(166)
Melatonin	MT2 receptor, HIF-1 pathway	Inhibits mast cell activation and pro-inflammatory cytokine release, reducing immune cell-mediated PCD and lung injury.	Approved (for sleep regulation)	No direct effect on viral replication; favorable safety profile.	(175, 176)
Indirect modulation of PCD (via viral replication inhibition)					
Oseltamivir	Neuraminidase (NA)	Inhibits viral release, reduces viral load, and indirectly mitigates PCD activation.	Approved	Effective against IAV/IBV; resistance may emerge (e.g., NA H275Y mutation).	(177–180)
Zanamivir	Neuraminidase (NA)	Inhibits viral release, reduces viral load, and indirectly mitigates PCD activation.	Approved	Effective against oseltamivir-resistant strains; intravenous formulation available for severe cases.	(181–186)
Baloxavir marboxil	PA (Endonuclease)	Inhibits viral mRNA synthesis, blocks replication, and indirectly reduces PCD activation.	Approved	Effective against IAV/IBV; resistance may emerge (e.g., PA I38X mutation).	(187–190)
Favipiravir	PB1	Induces lethal mutations in viral RNA, inhibits replication, and indirectly reduces PCD activation.	Approved/Emergency Use Authorization	Broad-spectrum activity against RNA viruses; teratogenic risk.	(191–193)
PROTACs (Representative strategy)	Viral proteins (e.g., HA, NA, PA) + E3 ligase	Specifically degrades viral proteins via the ubiquitin-proteasome pathway, fundamentally inhibiting viral replication and indirectly reducing virus-induced PCD.	Preclinical research	High barrier to resistance; potential broad-spectrum activity; long-lasting effects.	(194–199)
Arbidol	HA	Inhibits viral membrane fusion, reduces viral entry, and indirectly decreases viral load and PCD.	Approved (in China and Russia)	Targets conserved region of HA; low resistance rate.	(200–202)
Nitazoxanide	HA	Inhibits HA maturation and viral assembly, indirectly reducing viral release and PCD.	Approved (for antiparasitic use)	Requires early administration; limited efficacy in severe cases.	(203–205)
HNC042	NA	Inhibits NA activity and prevents viral release; effective against oseltamivir-resistant strains.	Phase I clinical trials	Designed to address oseltamivir resistance.	(206)
AV5080	NA	Inhibits NA activity and prevents viral release.	Phase III clinical trials	Effective against various IAV strains (including resistant ones); favorable oral properties.	(207)
CD388	NA + Fc receptor	Conjugate that combines NA inhibition and Fc-mediated immune effects, indirectly reducing PCD.	Phase II clinical trials	Long-acting; potential synergistic effects; reduced resistance risk.	(206)
Radavirsen	M1/M2 mRNA	Antisense oligonucleotide that inhibits M1/M2 protein translation, impairs viral assembly, and indirectly reduces PCD.	Phase I clinical trials	Effective only against IAV.	(208, 209)
TCN-032	M2e	Monoclonal antibody that inhibits viral spread by blocking budding and mediating immune effects, indirectly reducing PCD.	Phase II clinical trials	Targets a conserved epitope; low resistance risk; ineffective against IBV.	(210, 211)
Pimodivir	PB2 (Cap-binding domain)	Inhibits “cap-snatching,” blocks viral mRNA synthesis, and indirectly reduces PCD.	Phase II clinical trials (monotherapy discontinued)	Synergistic with oseltamivir; effective only against IAV.	(212–215)
ZSP1273	PB2 (Cap-binding domain)	Inhibits “cap-snatching,” blocks viral mRNA synthesis, and indirectly reduces PCD.	Phase III clinical trials	Effective against IAV; potential broad-spectrum activity; low resistance risk.	(163, 216)
CC-42344	PB2 (Cap-binding domain)	Inhibits “cap-snatching,” suppresses viral replication, and indirectly reduces PCD.	Phase II clinical trials	Effective against multiple IAV strains; potential use in pandemics.	(217)
GP681	PA (Endonuclease)	Prodrug that inhibits PA endonuclease activity, suppresses viral replication, and indirectly reduces PCD.	Phase III clinical trials	Effective against IAV/IBV; potential efficacy against resistant strains.	(218–220)
ZX-7101	PA (Endonuclease)	Prodrug that inhibits PA endonuclease activity, blocks viral mRNA synthesis, and indirectly reduces PCD.	Phase I clinical trials	*In vitro* activity comparable to baloxavir; protective *in vivo*.	(221)
AL-794	PA (Endonuclease)	Prodrug that inhibits PA endonuclease activity, blocks viral mRNA synthesis, and indirectly reduces PCD.	Phase I clinical trials (Discontinued)	No effective and well-tolerated dose identified.	(222, 223)
TG-1000	PA (Endonuclease)	Prodrug that inhibits PA endonuclease activity, suppresses viral replication, and indirectly modulates PCD.	Phase II clinical trials	Effective against IAV/IBV and oseltamivir-resistant strains.	(224)

The first comprises cell death inhibitors, which aim to block programmed cell death pathways such as necroptosis and pyroptosis, thereby alleviating IAV-induced tissue damage and excessive inflammation. The second involves agents that enhance cellular tolerance, enabling host cells to better withstand viral infection and PCD-inducing signals, thus reducing pathological cell death and preserving tissue homeostasis. The third strategy includes viral protein–targeting agents, such as compounds that inhibit or degrade viral components like HA and NA, thereby suppressing viral replication and release and indirectly limiting PCD activation. Nevertheless, some conventional antivirals already in clinical use—such as the neuraminidase inhibitor oseltamivir—remain constrained by rapid emergence of resistant variants and limited broad-spectrum efficacy. Consequently, there is an urgent need to develop more effective therapeutic approaches that precisely target PCD-related mechanisms in IAV infection.

Notably, combining novel PCD-targeting candidates with conventional antivirals presents both potential synergistic advantages and specific challenges. Traditional virus-targeted antivirals primarily act by reducing viral load and replication, thereby indirectly attenuating downstream PCD activation. In contrast, host-directed PCD modulators act on pathological pathways downstream of viral replication, and may preserve tissue integrity even in the presence of residual virus, potentially improving disease outcomes. This mechanistic complementarity provides a theoretical rationale for combination therapy. However, premature or excessive suppression of inflammatory PCD during the early phase of uncontrolled viral replication could impair antiviral immune clearance, particularly under conditions of incomplete viral inhibition, highlighting the need for careful assessment of potential antagonistic effects. Thus, the timing of administration and selection of appropriate patient populations are critical considerations in combination strategies.

Furthermore, protein degradation-targeting approaches, exemplified by proteolysis-targeting chimeras (PROTACs), further expand therapeutic possibilities (196). These strategies induce ubiquitin–proteasome-mediated degradation of viral proteins such as HA, NA, or PA, inhibiting viral replication at multiple stages and indirectly alleviating virus-induced PCD. Because PROTACs rely on host endogenous degradation machinery rather than direct enzymatic inhibition, they present a higher barrier to resistance and are mechanistically compatible with existing antiviral agents. Overall, rational integration of virus-targeted drugs with PCD modulation strategies requires systematic evaluation of mechanistic synergy, immunological consequences, and optimal intervention timing to maximize clinical benefit.

Despite recent advances in elucidating the molecular mechanisms and clinical implications of PCD during IAV infection, several critical knowledge gaps remain. The determinants that drive preferential activation of specific PCD pathways in distinct infection contexts are not fully defined, and how viral load, strain-specific features, host genetic background, and cellular metabolic status collectively shape the death fate of infected cells remains to be systematically clarified. Additionally, the precise spatiotemporal organization of PCD signaling cascades during infection is poorly understood. It remains unclear whether distinct death signals are activated sequentially, in parallel, or undergo context-dependent modulation at the single-cell or population level-particularly across different respiratory epithelial subtypes and immune cell populations under diverse stress conditions. Addressing these gaps will require integrated application of single-cell and spatial multi-omics, systems biology modeling, and artificial intelligence-assisted pathway node prediction. Comprehensive insights into these questions will provide a theoretical foundation for the rational integration of virus-targeted antivirals with host-directed PCD modulation or disease-tolerance strategies, enabling optimization of intervention timing, minimization of potential immunological antagonism, and design of combination therapies that concurrently limit viral replication, preserve epithelial barrier integrity, and mitigate immunopathology.

## Conclusion

Overall, distinct forms of PCD do not operate in isolation but instead constitute a highly integrated and dynamically regulated network, characterized by extensive interconnectivity, crosstalk, and compensatory mechanisms among individual pathways. The modes of PCD activation and their ultimate outcomes exert profound spatiotemporal influences on host immune responses, the extent of tissue injury, and overall disease prognosis. A systematic dissection of the regulatory logic governing the PCD network and its functional relevance during IAV infection will facilitate a conceptual shift from monolithic antiviral strategies toward multi-target, mechanistically integrated therapeutic paradigms, thereby providing a robust theoretical foundation for the development of more effective and safer anti-influenza interventions.
